# Thioredoxin interacting protein (TXNIP) is a novel tumor suppressor in thyroid cancer

**DOI:** 10.1186/1476-4598-13-62

**Published:** 2014-03-19

**Authors:** Jennifer A Morrison, Laura A Pike, Sharon B Sams, Vibha Sharma, Qiong Zhou, Jill J Severson, Aik-Choon Tan, William M Wood, Bryan R Haugen

**Affiliations:** 1 Department of Medicine, Division of Endocrinology, Diabetes, & Metabolism, University of Colorado Anschutz Medical Campus, Aurora, Colorado, USA; 2 Department of Pathology and University of Colorado Cancer Center, University of Colorado, Aurora, Colorado, USA; 3 Department of Pharmaceutical Sciences, University of Colorado Anschutz Medical Campus, Aurora, Colorado, USA; 4 University of Colorado Cancer Center and Department of Medicine, Division of Medical Oncology, University of Colorado Anschutz Medical Campus, Aurora, Colorado, USA

**Keywords:** Thyroid cancer, Thioredoxin interacting protein, TXNIP, Tumor suppressor, Orthotopic model, PPARγ

## Abstract

**Background:**

Thyroid cancer is the most common endocrine malignancy, and many patients with metastatic differentiated thyroid cancer (DTC), poorly differentiated thyroid cancer (PDTC), and anaplastic thyroid cancer (ATC) fail to respond to conventional therapies, resulting in morbidity and mortality. Additional therapeutic targets and treatment options are needed for these patients. We recently reported that peroxisome proliferator-activated receptor gamma (PPARγ) is highly expressed in ATC and confers an aggressive phenotype when overexpressed in DTC cells.

**Methods:**

Microarray analysis was used to identify downstream targets of PPARγ in ATC cells. Western blot analysis and immunohistochemistry (IHC) were used to assess thioredoxin interacting protein (TXNIP) expression in thyroid cancer cell lines and primary tumor specimens. Retroviral transduction was used to generate ATC cell lines that overexpress TXNIP, and assays that assess glucose uptake, viable cell proliferation, and invasion were used to characterize the *in vitro* properties of these cells. An orthotopic thyroid cancer mouse model was used to assess the effect of TXNIP overexpression in ATC cell lines *in vivo*.

**Results:**

Using microarray analysis, we show that TXNIP is highly upregulated when PPARγ is depleted from ATC cells. Using Western blot analysis and IHC, we show that DTC and ATC cells exhibit differential TXNIP expression patterns. DTC cell lines and patient tumors have high TXNIP expression in contrast to low or absent expression in ATC cell lines and tumors. Overexpression of TXNIP decreases the growth of HTh74 cells compared to vector controls and inhibits glucose uptake in the ATC cell lines HTh74 and T238. Importantly, TXNIP overexpression in T238 cells results in attenuated tumor growth and decreased metastasis in an orthotopic thyroid cancer mouse model.

**Conclusions:**

Our findings indicate that TXNIP functions as a tumor suppressor in thyroid cells, and its downregulation is likely important in the transition from differentiated to advanced thyroid cancer. These studies underscore the potential of TXNIP as a novel therapeutic target and prognostic indicator in advanced thyroid cancer.

## Background

Thyroid cancer is the most common endocrine malignancy, and it is estimated that nearly 63,000 new cases of thyroid cancer will be diagnosed in the United States in 2014 [1]. Although the majority of these patients have well-differentiated thyroid cancer and respond favorably to conventional therapies (surgery with or without radioactive iodine I^131^ therapy and suppression therapy with thyroid hormone), a significant minority of patients develops advanced disease that is resistant to standard treatments. For the minority of individuals who develop anaplastic thyroid cancer (ATC), an aggressive and undifferentiated form of thyroid cancer, the prognosis is very poor and median survival is 3–5 months [2,3]. The mechanisms underlying the development of poorly differentiated thyroid cancer (PDTC) and ATC are incompletely understood. Therapeutic options for these patients are limited, and prognosis remains dismal. This is an area for which further research and drug/therapy development is critically needed.

We recently reported that peroxisome proliferator-activated receptor gamma (PPARγ) confers an aggressive phenotype in thyroid cancer cells [4]. Nuclear PPARγ expression was absent in differentiated thyroid cancer (DTC) cell lines and high in ATC cell lines. When PPARγ was overexpressed in the DTC cell line BCPAP, *in vitro* proliferation and invasive capacity were increased. Furthermore, when PPARγ was depleted from ATC cells, *in vitro* proliferation and invasive capacity were inhibited and tumor growth was inhibited in two *in vivo* murine cancer models (an orthotopic thyroid model and a flank xenograft model). These data challenge the widely-held assumption that PPARγ is a tumor suppressor and suggest that PPARγ may mediate the aggressive phenotype that develops as part of the transition from DTC to PDTC and ATC.

In the studies presented here, we show that *TXNIP*, the gene encoding thioredoxin interacting protein (TXNIP), is a negatively-regulated downstream target of PPARγ. TXNIP is a negative regulator of cell growth and metabolism [5-11]. It modulates cellular redox status by binding to and inhibiting thioredoxin, a principal component of the cell’s antioxidant system [12-14]. Furthermore, via its negative regulation of thioredoxin, TXNIP can inhibit invasion and metastasis and promote a pro-apoptotic cellular environment [15-20]. TXNIP has been shown to be a tumor suppressor in cancer [8,21-28], but its role in thyroid cells or in thyroid cancer has not been investigated. In this current study, we show that TXNIP is highly expressed in DTC and low or undetectable in ATC and appears to be a novel tumor suppressor in thyroid cancer.

## Results

### TXNIP is upregulated in PPARγ-depleted ATC cells

**Figure 1:**
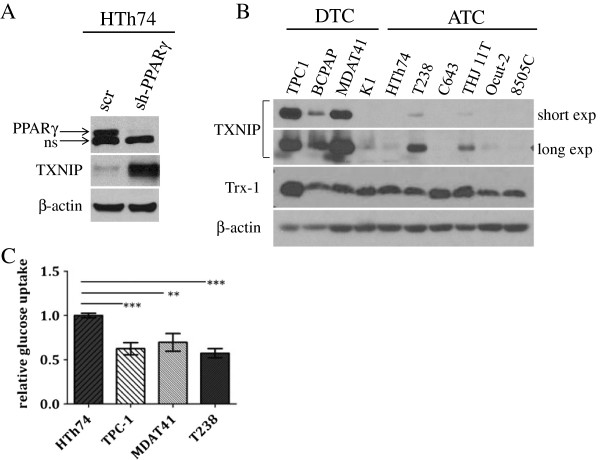
**TXNIP is highly expressed in DTC cell lines and low or undetectable in ATC cell lines, and glucose uptake is inversely proportional to TXNIP expression levels. (A)** HTh74 cells were transduced with lentivirus expressing a PPARγ-specific shRNA or scrambled control, and stable pools were generated under antibiotic selection. Western blot analysis of nuclear lysates (top immunoblot) reveals PPARγ expression levels in the transduced cells. Using Western blot analysis of whole cell lysates, TXNIP and β-actin (loading control) were detected using specific antibodies. Nonspecific band is indicated by “ns”. **(B)** Western blot analysis for TXNIP, Trx-1, and β-actin (loading control) were performed on whole cell lysates prepared from a panel of DTC and ATC cell lines grown under standard conditions. **(C)** Glucose uptake assays were performed. Each condition was performed in triplicate per experiment, and each experiment was performed at least three times. Nonspecific glucose uptake as determined by parallel treatment of a subset with cytochalasin B was subtracted from measurements. Data from all experiments were combined, and glucose uptake from each cell line was normalized to levels of HTh74 (average set at 1). Normalized averages are plotted, and error bars indicate SEM. ***p <0.0001, **p <0.001.

We previously reported that PPARγ confers an aggressive phenotype in thyroid cancer cells [4]. To investigate downstream mediators of these effects, a discovery-based microarray approach was used to compare mRNA expression levels in HTh74 ATC cells expressing a PPARγ-specific shRNA compared to a scrambled control. The most highly up- and down-regulated genes are shown in Table 1. *PPARG* was the most highly downregulated gene, which is consistent with the use of a PPARγ-specific shRNA. The most highly upregulated gene in the PPARγ-depleted HTh74 cells was thyroid peroxidase, an enzyme critical to synthesis of thyroid hormone. Interestingly, *TXNIP* was the second most highly upregulated gene in the PPARγ-depleted ATC cells (>10-fold). TXNIP is a known tumor suppressor whose role in thyroid cancer has never been reported. Western blot analysis of whole cell extracts confirms that TXNIP is also upregulated at the protein level when PPARγ expression is knocked down in the HTh74 ATC cells (Figure 1A). These data imply that TXNIP downregulation in HTh74 cells is a downstream consequence of high nuclear PPARγ expression, as TXNIP expression is increased when HTh74 cells are depleted of PPARγ. These data are consistent with a previously published report that the TXNIP promoter contains multiple PPARγ binding sites and that TXNIP expression is negatively regulated by binding of PPARγ to the TXNIP promoter and by treatment with PPARγ agonists [29].

**Table 1:** List of highly upregulated and downregulated genes when PPARγ expression is depleted from the HTh74 ATC cell line

**Gene expression changes in PPARγ-depleted HTh74 cells**		
**Gene**	**Description**	**Fold change**
**Upregulated**		
*TPO*	Thyroid peroxidase	11.7
***TXNIP***	**Thioredoxin interacting protein**	**10.7**
*C1orf168*	Chromosome 1 open reading frame 168	10.2
*NFASC*	Neurofascin homolog (chicken)	7.6
*SFRP4*	Secreted frizzled-related protein 4	7.6
*LOC727770*	Similar to ankyrin repeat domain 20 family, member A1	7.2
*ASTN1*	Astrotactin 1	7.1
**Downregulated**		
***PPARG***	**Peroxisome proliferator-activated receptor gamma**	**−9.2**
*EVI2B*	Ecotropic viral integration site 2B	−7.5
*LOC643201*	Hypothetical protein LOC643201	−7.2
*NQO1*	NAD(P)H dehydrogenase, quinone 1	−7.1
*ACTG2*	Actin, gamma 2, smooth muscle, enteric	−6.3
*ARHGAP26*	Rho GTPase activating protein 26	−6.0
*RLN2*	Relaxin 2	−5.7

Stable cell lines were generated via transduction of the ATC cell line HTh74 with lentivirus encoding PPARγ-specific shRNA or scrambled control followed by selection in puromycin 0.5 μg/mL. Prepared RNA was then subjected to microarray analysis. Genes whose mRNA levels varied by greater than 5-fold are shown in the table along with fold change.

### TXNIP is expressed at high levels in DTC cells and tissues compared with ATC, and TXNIP expression correlates inversely with glucose uptake

We next investigated whether there is differential TXNIP expression in DTC versus ATC cells. Western blot analysis revealed high TXNIP expression in the DTC cell lines TPC-1, MDA-T41, and BCPAP in contrast to low or undetectable expression in the ATC cell lines HTh74, C643, Ocut-2, and 8505C (Figure 1B). This disparate TXNIP expression between DTC and ATC cell lines, however, was not absolute. The ATC cell lines T238 and TJH11T had levels of TXNIP expression comparable to some DTC cells, whereas the DTC cell line K1 had lower TXNIP expression in comparison to the other DTC cell lines. Similar trends were observed at the mRNA level (data not shown).

TXNIP is a known binding partner and inhibitor of the cellular antioxidant and tumor promoter thioredoxin (Trx) [12,14]. Thioredoxin may be localized to the cytoplasm or nucleus (Trx-1) where it can function as a cofactor, detoxify reactive oxygen species, or modulate the activity of transcription factors, or to mitochondria (Trx-2), and high Trx-1 expression is associated with more aggressive disease in other cancer types [30-36]. As TXNIP is a negative regulator of Trx and disparate TXNIP expression was observed between the panels of DTC and ATC cell lines, we sought to identify differences in Trx-1 expression patterns between the two groups. Immunoblot analysis revealed no clear differences in Trx-1 expression levels between DTC and ATC (Figure 1B).

TXNIP is also a known inhibitor of glucose uptake [5,7,11]. We, therefore, investigated whether differential TXNIP expression correlates with differences in glucose uptake. Analysis of glucose uptake of two ATC and two DTC cell lines was performed, and the ATC cell line HTh74, which has the lowest TXNIP levels of the examined cell lines, had the highest level of glucose uptake (Figure 1C). Interestingly, the ATC cell line T238 had glucose uptake at levels comparable to that observed with the two DTC cell lines TPC-1 and MDA-T41. This finding is consistent with a higher level of TXNIP expression in this cell line (Figure 1B).

**Figure 2:**
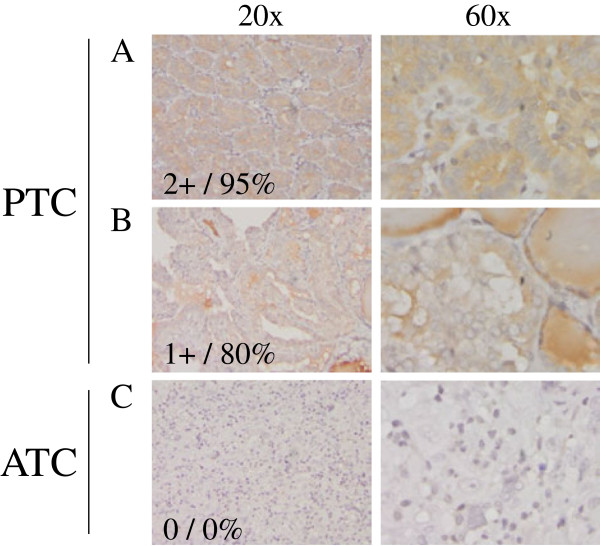
**TXNIP is expressed in primary DTC tumors but is low or undetectable in ATC tumors.** Immunohistochemistry was performed using a TXNIP-specific primary antibody or normal mouse IgG control and a horseradish peroxidase tagged secondary antibody on a panel of 13 PTC and 8 ATC patient specimens. Two representative PTC **(A-B)** and one ATC **(C)** specimen stains are shown at 20X and 60X power. Positive staining is indicated by brown coloring, though staining of colloid is nonspecific. Staining was quantitated by a pathologist (S. B. S.), and intensity of staining was scored from absent (0) to high to (3+), followed by percent of tumor positivity.

We next investigated whether TXNIP was differentially expressed at the tumor tissue level. Immunohistochemistry to detect TXNIP protein expression was performed on paraffin-embedded tumor blocks of 13 well-differentiated primary papillary thyroid cancer (PTC) tumors and 8 ATC patient specimens. Representative images at high and low magnification are shown for PTC (Figure 2A-B) and ATC (Figure 2C). TXNIP expression was undetectable in 63% of ATC specimens in contrast to 15% of PTC specimens, though there was one ATC specimen that exhibited high TXNIP staining. This differential TXNIP expression between PTC and ATC is similar to the pattern observed with the thyroid cancer cell lines (Figure 1B). Therefore, loss of or absence of TXNIP expression appears to correlate with more aggressive thyroid cancer in cell lines and tumor tissue.

### TXNIP overexpression in the ATC cells attenuates *in vitro* glucose uptake and growth

**Figure 3:**
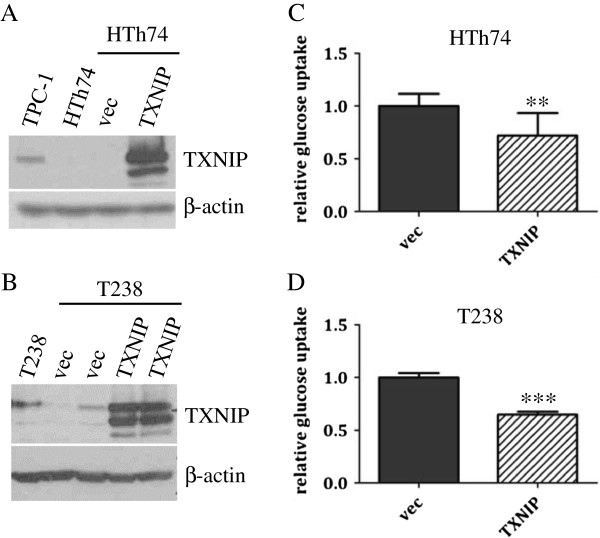
**TXNIP overexpression in ATC HTh74 and T238 cells attenuates glucose uptake.** HTh74 and T238 cells were transduced with retrovirus encoding human TXNIP or vector control as well as a selectable antibiotic resistance marker, and stable pools were generated under antibiotic selection. Western blot analysis of whole cell lysates with TXNIP- and β-actin-specific antibodies is shown for HTh74 **(A)** and T238 **(B)**. Glucose uptake assays were performed as described in Figure 1 using the HTh74 stable cell lines **(C)** and T238 stable cell lines **(D)**. Data from all experiments were combined, and glucose uptake from each cell line was normalized to vector control levels (average set at 1). **p = 0.001, ***p <0.0001.

Based on its differential expression in DTC and ATC, we predicted that TXNIP acts as a tumor suppressor in thyroid cells and that its downregulation plays an important role in the development of an aggressive thyroid cancer phenotype. To further investigate this hypothesis, we re-expressed TXNIP in ATC cell lines. HTh74 and T238 ATC cell lines were transduced with a retroviral vector encoding human TXNIP. Western blot analysis verified increased TXNIP expression in the transduced HTh74 (Figure 3A) and T238 (Figure 3B) cell lines compared to control cells transduced with empty vector. As a functional read out of TXNIP expression, we assessed glucose uptake in the stable cell lines. TXNIP overexpression significantly inhibited glucose uptake in both the HTh74 and T238 cell lines (Figure 3C and 3D, respectively), consistent with its known function of glucose uptake inhibition in other tissues.

**Figure 4:**
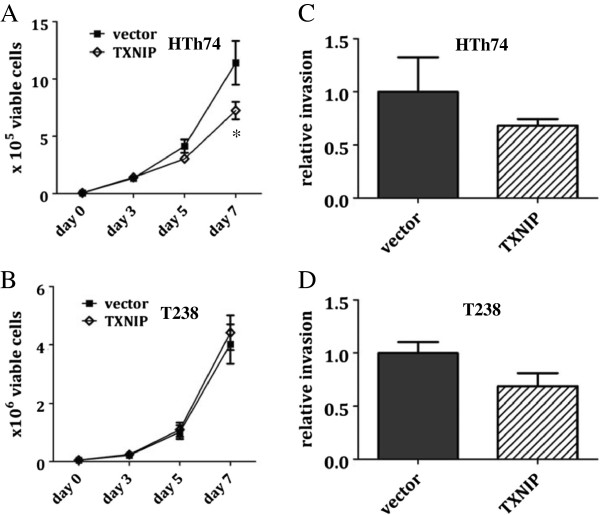
**TXNIP overexpression inhibits *in vitro *growth of ATC HTh74 cells.** Viable cell proliferation assays were performed for stable HTh74 **(A)** and T238 **(B)** cell lines. Briefly, 50,000 cells were plated in 6-cm plates, trypsinized and viable cell counts were determined using the ViCell automated cell counting system. Each time point was performed in duplicate, and lysates were prepared from the day 7 time point to confirm high TXNIP expression using Western blot analysis. Each experiment was performed at least three times. Mean and SEM are plotted. Closed squares (∎) indicate vector control cells, and open diamonds (◊) indicate cells with TXNIP overexpression. *In vitro* invasion assays were performed on the TXNIP-overexpressing stable HTh74 **(C)** and T238 **(D)** cell lines as described in the Methods section. Results from three independent experiments were combined and normalized to the vector control average and graphed with mean plus SEM. *p <0.01 (Figure 4A), Figure 4B p = 0.9021, Figure 4C p = 0.3523, Figure 4D p = 0.0754.

To assess the effect of TXNIP overexpression on the growth of these ATC cell lines, viable cell proliferation assays were performed under standard growth conditions. TXNIP overexpression resulted in slowed growth of HTh74 cells by 37% (Figure 4A). Interestingly, however, TXNIP overexpression in T238 cells had no effect on the *in vitro* growth rate (Figure 4B). The reasons for the observed differences in TXNIP-mediated growth effects between these two cell lines are unclear, however, there are a few potential causes or factors that might contribute to this discrepancy. The baseline proliferation rate of parental T238 cells is much higher than parental HTh74 cells (data not shown), and this may obscure our ability to detect more subtle growth inhibitory effects with TXNIP overexpression in the T238 cell line. Alternatively, differential pathway activation might explain the *in vitro* growth differences between T238 and HTh74 cells and the ability of T238 cells to circumvent TXNIP-mediated growth deceleration. The T238 parental cell line has some basal TXNIP expression, and these cells have likely acquired the ability to resist some of the *in vitro* growth inhibitory effects of TXNIP via other mechanisms. In addition, the standard growth media used in propagation of these cell lines provides supplemental glutamine and glucose, and this enriched nutrient media may permit T238 cells to overcome the metabolic inhibitory effects of TXNIP overexpression *in vitro*. These potential limitations of *in vitro* assay systems, which do not adequately recapitulate *in vivo* tumor conditions, underscore the importance of further evaluation in an *in vivo* model system. In addition to proliferation assays, we performed invasion assays using an *in vitro* Matrigel invasion model [37], and TXNIP overexpression resulted in a trend towards decreased invasion in both cell lines but this did not reach statistical significance (Figure 4C-D).

### TXNIP expression in ATC cells results in attenuated tumor growth and metastasis in an *in vivo* orthotopic thyroid cancer mouse model

**Figure 5:**
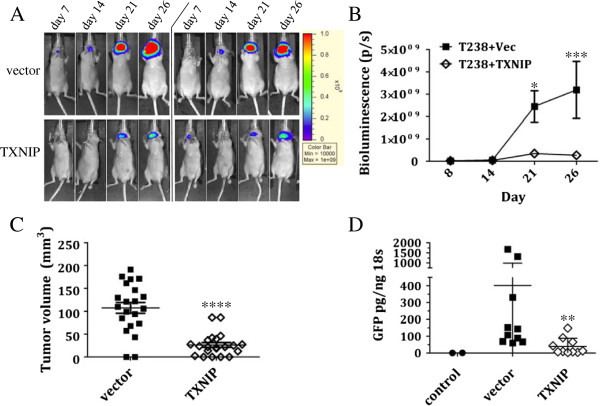
**TXNIP overexpression in ATC T238 cells attenuates tumor growth and metastasis in an *in vivo *orthotopic murine thyroid cancer model.** T238 cells stably expressing luciferase-IRES-GFP and either TXNIP or empty vector were injected into the right lobe of the thyroid gland of athymic nude mice with the aid of a dissecting microscope to enhance visualization. Weekly imaging with IVIS after injection of luciferin was performed to monitor tumor establishment and growth. There were 10–11 mice per group in each experiment, and the experiment was performed two times. **(A)** Representative images of one mouse per group imaged by IVIS over time are shown. **(B)** Quantitation of the bioluminescence from one experiment is shown with average and SEM. Closed squares (∎) indicate vector control group, and open diamonds (◊) indicate TXNIP group. **(C)** Final tumor volumes, as calculated from caliper measurements, from both experiments are combined and plotted with averages and SEM. To assess for lung metastasis, RNA isolated from whole lungs was subjected to qRT-PCR analysis to assess GFP expression. Data was normalized to 18 s RNA levels, and averages plus SD were plotted **(D)**. RNA isolated from lungs of mice receiving no tumor cell injections was included as an additional negative control for GFP expression and is indicated with closed circles (●). *p <0.05, **p =0.0021, ***p <0.001, ****p <0.0001.

Finally, we examined the effect of TXNIP overexpression in an *in vivo* orthotopic tumor model. The orthotopic thyroid cancer mouse model is a well-established model that closely mimics the features of human thyroid cancer with regard to growth and metastases than does the more commonly-used subcutaneous flank xenograft model [4,38-41]. Tumor cells expressing luciferase-IRES-GFP were injected into the right thyroid lobe and monitored weekly by IVIS imaging for tumor establishment and growth. TXNIP overexpression in the T238 cell line resulted in attenuated bioluminescence compared to vector control (Figure 5A-B), and resultant tumor volumes were significantly smaller (Figure 5C). Though there was a significant attenuation in bioluminescence as well as a trend toward smaller tumors with the TXNIP-expressing HTh74 cells compared to vector controls, the final tumor volumes were not significantly different (data not shown).

In our experience, T238 cells in this orthotopic model frequently result in lung metastases (unpublished data). To determine if TXNIP overexpression affected distant metastatic spread in this orthotopic model, lungs were collected at necropsy and snap-frozen, homogenized, and RNA was subsequently isolated and subjected to qRT-PCR to detect GFP expression, a marker of lung metastases in this model. Metastatic tumor burden was significantly reduced in the mice injected with TXNIP-overexpressing T238 cells compared to vector controls (Figure 5D). Control mice with no tumor cells injected had no detectable GFP mRNA in their lung tissues.

## Discussion

In this report, we identified TXNIP as a novel tumor suppressor in thyroid cancer. TXNIP is highly upregulated when PPARγ, which we have previously shown to be a tumor promoter [4], is depleted from ATC cells. Furthermore, DTC cell lines and primary PTC tumors have high endogenous TXNIP levels, whereas TXNIP expression is low or absent in ATC cell lines and primary tumor specimens. This TXNIP expression pattern is opposite to what we previously reported with PPARγ, which is highly expressed in ATC cell lines and absent in DTC cell lines and whose forced expression confers a more aggressive phenotype in thyroid cancer cells *in vitro* and *in vivo*[4]. These data are consistent with a previously published report that TXNIP is a negatively-regulated target of PPARγ [29]. Therefore, TXNIP expression appears to be downregulated or lost in the progression from well-differentiated thyroid tumors to more aggressive, undifferentiated tumors. TXNIP has been shown to be a tumor suppressor in other cell types, and our results here show for the first time that it serves a similar function in thyroid cells.

The novel finding that TXNIP expression is lost in the progression from well-differentiated PTC to undifferentiated, aggressive ATC is consistent with our hypothesis that TXNIP is a tumor suppressor in thyroid cancer. Patients with well-differentiated PTC respond well to conventional therapy and have an excellent overall survival rate. Poorly-differentiated PTC tumors that have lost the ability to concentrate iodine often fail to respond to conventional therapy and result in poorer outcomes, and undifferentiated ATC portends an extremely-poor prognosis and is generally fatal within 3–5 months [2,3]. The apparent loss of TXNIP expression during the progression from well-differentiated to poorly-differentiated and undifferentiated thyroid cancer is consistent with its role as a tumor suppressor in thyroid cells. Loss of TXNIP expression has been reported to correlate with more aggressive disease, advanced stage, and poorer prognosis in breast, gastric, colorectal, and bladder cancers, as well as diffuse large B-cell lymphoma [23,24,42-47], and TXNIP mRNA expression has been shown to be inversely proportional to melanoma progression [27]. TXNIP expression in cancer may be downregulated through epigenetic, transcriptional, post-transcriptional, or translational mechanisms (reviewed by Zhou *et al.*[21]). Though the tumor promoter and cellular antioxidant Trx-1, which is inhibited by TXNIP, has been shown to confer more aggressive disease in other cancers [30-34], our data failed to show differences in Trx-1 expression levels between DTC and ATC cells.

Overexpression of TXNIP in the HTh74 ATC cell line resulted in slowed *in vitro* growth. This negative growth regulatory effect has been seen in other systems as well. TXNIP overexpression in the human gastric carcinoma cell lines AGS, SNU-16, and SNU-620, the promyelocytic leukemia cell line HL-60, and HTLV-I-positive T cells led to growth reduction *in vitro*[8,22,48]. Lung fibroblasts from TXNIP knockout mice proliferate at a faster rate than wild-type, implying that loss of TXNIP promotes or allows for enhanced proliferation [9]. Interestingly, we did not observe an *in vitro* growth inhibitory effect in the T238 cell line, which has some basal endogenous TXNIP expression. It is likely that the effects of TXNIP on cell growth and proliferation are cell-context dependent and might be circumvented through activation of alternative mitogenic pathways. Furthermore, *in vitro* cell culture conditions do not adequately recapitulate the tumor microenvironment and contributions of paracrine-mediated signaling, underscoring the importance of *in vivo* studies. In accordance with this potential limitation, Goldberg and colleagues failed to see slowed *in vitro* growth of melanoma cells transfected with TXNIP though when injected in an orthotopic flank model in nude mice, slowed tumor growth/development was observed [27]. Although we observed a trend toward decreased invasion by TXNIP overexpression in two ATC cell lines, this effect did not reach statistical significance in our *in vitro* model.

Importantly, in a well-established orthotopic murine thyroid cancer model that mimics human thyroid cancer with regard to growth and metastasis, we show that TXNIP overexpression in the ATC T238 cell line resulted in significant attenuation of both tumor growth and pulmonary metastatic burden. These data support our hypothesis that TXNIP is a tumor suppressor in thyroid cells. TXNIP has been shown to be a tumor suppressor in other animal models of cancer as well. A mouse strain with a spontaneous nonsense mutation in *TXNIP* has dramatically increased incidence of spontaneous hepatocellular carcinomas (HCC) [25], and TXNIP-knockout mice develop increased number and size of HCC in a diethylnitrosamine (DEN)-induced murine model of HCC [28]. In a murine gastric carcinoma model in which tumors are induced via infection with *Helicobacter pylori* and cotreatment with *N*-methyl-*N*-nitrosourea, concomitant knock out of TXNIP resulted in increased numbers of tumors, heightened preneoplastic changes, increased percentage of malignant tumors, and elevated inflammatory marker expression compared to control mice with wild-type TXNIP expression [22]. In a murine model of bladder cancer in which tumors are induced by treatment with *N*-butyl-*N*-(4-hydroxybutyl) nitrosamine (BBN), genetic deletion of TXNIP results in accelerated development of high grade and invasive tumors by ~4 weeks compared to controls with wild-type expression, however, controls eventually succumb to tumor development and TXNIP expression in these tumors has been downregulated by other mechanisms [24].

In the orthotopic ATC model, TXNIP overexpression also led to a significant reduction in pulmonary metastatic burden. Inhibition of metastasis conferred by TXNIP overexpression has been shown in other systems as well. B16F10 melanoma cells transfected with TXNIP then injected via tail vein into C57BL/6 mice resulted in decreased lung metastases [23]. TXNIP-transfected melanoma cells resulted in fewer metastases in both a nude mouse flank tumor model and IV tail injection metastasis model relative to vector controls [27]. In human breast cancer, high TXNIP levels are associated with longer metastasis-free intervals and better prognosis than those with low TXNIP expression [43,46]. These data implicate TXNIP as a tumor suppressor in a variety of cancers and, for the first time, is now shown to be a tumor suppressor in thyroid cells.

Curiously, TXNIP overexpression in the ATC cell line HTh74 resulted in reduced *in vitro* growth but no significant difference on *in vivo* growth in the orthotopic thyroid cancer model. Although bioluminescence signals were attenuated in the TXNIP-expressing HTh74 cells versus controls, final tumor volumes were not significantly different, though a trend toward smaller tumors with injection of the TXNIP-expressing HTh74 cells compared to vector controls was observed. TXNIP-overexpressing and vector control tumors did not look different histologically. In our prior studies using HTh74 cells in the orthotopic murine thyroid cancer model system, we have observed that the *in vivo* growth rates are slower compared to other thyroid cancer cell lines (84 days to achieve 100 mm^3^ tumors compared with 28–35 days in other ATC cell lines). It is possible that if our study had been temporally extended, the trend in tumor volume attenuation in the TXNIP-overexpressing group might have reached statistical significance. It is also possible that TXNIP does not play a significant *in vivo* role on malignant behavior in the HTh74 cells, but our *in vitro* data would suggest otherwise.

In keeping with the known function of TXNIP as a glucose uptake inhibitor, we showed that the degree of glucose uptake was inversely correlated with TXNIP levels in the examined thyroid cancer cell lines. An interesting aspect of thyroid cancer biology relates to its properties on 2-deoxy-2-fluoro-D-glucose positron emission topography computed topography (FDG PET/CT) imaging. FDG PET imaging can be negative in many patients with DTC and distant metastases, and this correlates with a relatively good prognosis in these patients [49,50]. Less differentiated PTC and ATC tumors are more likely to be PET positive, PET positive lesions are more likely to be resistant to conventional radioactive I^131^ treatment, and increased intensity of FDG uptake is associated with a poorer prognosis and increased mortality [49,50]. The mechanism underlying this differential glucose uptake between well-differentiated PTC and ATC is not well understood. The novel finding that TXNIP expression is low in ATC is consistent with the observed FDG uptake on PET/CT in patients with ATC, supporting a critical role for TXNIP as a metabolic regulator in thyroid cancer progression.

In addition to inducing a metabolic shift important to tumor biology, downregulation of TXNIP has other important effects in cancer cells that contribute to tumor promotion and/or progression. TXNIP can reduce tumor invasion and angiogenesis through inhibition of thioredoxin and can directly impact cell survival by promoting a pro-apoptotic environment [13,15-20]. Independent of its interaction with thioredoxin, TXNIP also has the ability to inhibit cell cycle progression by indirectly stabilizing the cell cycle inhibitor p27^Kip1^[9]. In addition, TXNIP indirectly inhibits mTOR, a regulator of cell growth and metabolism [6]. Therefore, downregulation of TXNIP in a tumor has the potential to promote cell survival, growth, invasion, and metastasis. The exact mechanisms by which TXNIP exerts its tumor suppressive functions in thyroid cancer cells are not yet clear. Future studies of the mechanisms by which TXNIP is expressed and functions in thyroid cancer will improve our understanding of the progression to advanced thyroid cancer and help to develop more effective targeted therapies.

## Conclusions

In conclusion, we report that TXNIP is a novel tumor suppressor in thyroid cancer. TXNIP is downregulated during the progression from well-differentiated thyroid cancers to poorly differentiated and anaplastic thyroid cancers. Overexpression of TXNIP in ATC cell lines resulted in slowed *in vitro* growth and glucose uptake inhibition. Importantly, in an *in vivo* orthotopic murine thyroid cancer model, TXNIP overexpression attenuated tumor growth and drastically diminished pulmonary metastatic tumor burden. These data highlight the importance of TXNIP as a potential therapeutic target and prognostic marker in advanced thyroid cancer.

## Methods

### Cell lines and maintenance

HTh74 and C643 cells were obtained from Dr. K. Ain (University of Kentucky, Lexington, KY) with permission from N. E. Heldin (University Hospital, Uppsala, Sweden). TPC1 cells were provided by S. Jhiang (Ohio State University, Columbus, OH). BCPAP and 8505C cells were provided by M. Santoro (Medical School, University of Naples Federico II, Naples, Italy). TJH11T cells were obtained from J. A. Copland (Mayo Clinic Comprehensive Cancer Center, Jacksonville, FL) and were maintained in RPMI 1640 supplemented with 10% fetal bovine serum (FBS), non-essential amino acids, 1 mM sodium pyruvate, 1 nM T3, 0.5 μg/mL hydrocortisone, 8 ng/mL epidermal growth factor, 25 mM HEPES, and 0.1 mg/mL Primocin. MDA-T41 cells were obtained from G. Clayman (University of Texas MD Anderson Cancer Center, Houston, TX). K1 cells were provided by D. Wynford-Thomas (Cardiff University, Cardiff, UK). T238 were obtained from L. Roque (Instituto Português de Oncologia, Lisboa, Portugal). Ocut-2 cells were obtained from N. Onoda (Osaka City University Graduate School of Medicine, Osaka, Japan). Except for TJH11T cells, all cell lines were maintained in RPMI 1640 supplemented with 5% FBS. All cells were passaged at 37°C in 5% CO_2_. Cell lines were authenticated by short tandem repeat (STR) profiling as previously described [51].

### PPARγ knockdown and microarray analysis

PPARγ-depleted HTh74 cells and scrambled control cells were generated as previously described using lentivirus expressing PPAR-specific shRNA or scrambled control [4]. Total RNA from PPARγ-depleted and scrambled control cells was isolated using an RNeasy Mini Kit (Qiagen) according to the manufacturer’s instructions. Integrity of the RNA preparation was verified on an Agilent Bioanalyzer 2100. Total RNA (5 μg) of each cell line was used for microarray analysis using the Human Genome U133 Plus 2.0 Array (Affymetrix), performed by the Gene Expression Core of the University of Colorado Denver, Anschutz Medical Campus (Aurora, CO). Gene expression profiles were normalized by robust multichip analysis (RMA), differentially expressed genes were analyzed by fold-change, using a cut-off of 2-fold, 122 and 198 genes were found to be up and down-regulated in the knockdown line. Enrichment analysis of the gene list was performed using Database for Annotation, Visualization and Integrated Discovery (DAVID) analysis software.

### Western blot analysis

Cells were trypsinized and lysed in extraction buffer (EB; 1% Triton X-100, 10 mM Tris pH 7.4, 5 mM ethylenediaminetetraacetic acid (EDTA), 50 mM sodium chloride (NaCl), 50 mM sodium fluoride, 2 mM sodium orthovanadate, and 1X cOmplete protease inhibitors [Roche Diagnostics]) and clarified by high speed centrifugation at 4°C. For nuclear PPARγ and Trx-1 expression determination, cells were fractionated into nuclear and cytosolic fractions using the Active Motif Nuclear Extract system, according to the manufacturer’s instructions. Whole cell and nuclear protein extracts (25 μg) were diluted in Laemmli sample buffer and resolved by sodium dodecyl sulfate polyacrylamide gel electrophoresis (SDS-PAGE) on 10% gels and transferred to polyvinylidene difluoride (PVDF) membranes (BioRad). Membranes were blocked for 1 hour at room temperature in 5% nonfat dry milk in 20 mmol/L Tris pH 7.4, 128 mmol/L NaCl, 0.1% Tween 20 (TBST), then incubated overnight in primary antibody at 4°C. Primary antibodies used for the current studies include anti-VDUP1 (TXNIP) at 1:500 (rabbit polyclonal; Invitrogen, catalog # 403700), anti-PPARγ at 1:500 (rabbit polyclonal; Santa Cruz Biotechnology, catalog # sc-7196), anti-thioredoxin-1 at 1:1,000 (C63C6 rabbit monoclonal; Cell Signaling Technology, catalog #2429), and anti-β-actin at 1:5,000 (mouse monoclonal; Sigma-Aldrich, catalog # A5441). After washing in TBST, membranes were incubated at room temperature for 1 hour in secondary antibodies conjugated to horseradish peroxidase (anti-rabbit at 1:5,000 for TXNIP, PPARγ, and thioredoxin-1 blots and anti-mouse at 1:10,000 for β-actin blots; GE Healthcare). SuperSignal West Pico Chemiluminescent substrate (Thermo Scientific) was used to detect immunoreactivity. Re-Blot Plus Mild (Millipore) was used to strip blots for purposes of reprobing with an alternate primary antibody.

### Immunohistochemistry

We retrospectively selected formalin-fixed, paraffin-embedded blocks of primary 13 PTC and 8 ATC specimens from the University of Colorado Hospital pathology archives for analysis of TXNIP protein expression by immunohistochemistry. Institutional review board approval was obtained. Sections were deparaffinized in Histoclear, rehydrated, and antigen retrieval in 10 mM sodium citrate buffer with 0.05% Tween 20, pH 6.0, was performed in a Biocare Medical decloaking chamber at 120°C for 5 minutes. Endogenous peroxidase activity was quenched by incubation in 3% hydrogen peroxide for 30 minutes at room temperature. Tissues were blocked with 5% goat serum in phosphate-buffered saline (PBS) with 1% bovine serum albumin for 1 hour at room temperature. Slides were incubated overnight at 4°C in primary anti-TXNIP antibody at a concentration of 1:400 (mouse monoclonal antibody IgG1, clone JY2, MBL International, catalog # K0205) diluted in antibody dilution buffer (0.01 M PBS, pH 7.2 with 0.05% sodium azide) or normal mouse IgG at an equivalent concentration as negative control (Santa Cruz Biotechnology, catalog # sc-2025). Slides were incubated in secondary goat anti-mouse antibody conjugated to horseradish peroxidase diluted in PBS at a concentration of 1:400 (Dako, catalog # P0447) for 1 hour at room temperature. For visualization, slides were incubated at room temperature for 2–4 minutes in ImmPACT 3, 3′-diaminobenzidine (DAB) peroxidase substrate (Vector Laboratories). Sections were counterstained in Mayer’s hematoxylin solution, dehydrated, dried, and mounted with Cytoseal (Thermo Scientific). Stained specimens were read and score by a pathologist (S. B. Sams) based on percent of specimen that stained positively and degree of intensity (0 to 3+).

### TXNIP overexpression

A plasmid encoding human TXNIP (pcDNA3.1-hTXNIP) was a kind gift from P. Patwari (Brigham and Women’s Hospital, Boston, MA). The TXNIP coding sequence was amplified via polymerase chain reaction (PCR), using primers TXNIP 1 F [5′ TAG CGG CCG CAT GGT GAT GTT CAA GAA GAT CAA GT 3′] and hTXNIP EcoRI rev [5′ GCG AAT TCT CAC TGC ACA TTG TTG TTG AGG A 3′], which added a *NotI* restriction site to the 5′ end and an *EcoRI* site on the 3′ end of the coding sequence, respectively. PCR product was ligated into the pCR2.1 shuttling vector (Invitrogen), excised via *NotI* and *EcoRI* digestion, gel purified, and directionally inserted into the retroviral vector pQCXIP (gift of S. Nordeen, University of Colorado Denver, Aurora, CO) pre-digested with *NotI* and *EcoRI*. Resultant pQCXIP-hTXNIP clones were confirmed with sequencing. Retrovirus was generated in the BOSC cell line [52] after transfection of pQCXIP or pQCXIP-hTXNIP with pCL-Ampho packaging vector (gift of H. Ford, University of Colorado Denver, Aurora, CO) using FuGENE 6 transfection reagent (Roche). Supernatant with virus was collected at 48 and 72 hours after transfection, centrifuged at low speed, filtered through 0.45 μm syringe filter (Fisher Scientific), and stored at −80°C. Anaplastic HTh74 and T238 cells were transduced with virus-containing supernatant mixed 1:1 with growth media and supplemented with 8 μg/mL polybrene (Sigma-Aldrich), as previously described [4]. In the vector pQCXIP, the coding sequence for the insert (TXNIP) is cotranscribed with a puromycin resistance gene as a bicistronic message via an internal ribosome entry site. Forty-eight hours after transduction, the cells were placed under selection in puromycin (Sigma-Aldrich) at a concentration of 0.5 μg/mL for HTh74 cells and 2.5 μg/mL for T238 cells as previously determined by kill curves.

### Glucose uptake assays

Cells were grown in 12-well plates with each condition plated in triplicate. Prior to glucose uptake determination, cells were rinsed in PBS, then incubated in low glucose DMEM without serum for 4 hours at 37°C. Cells were then incubated in Krebs buffer (140 mM NaCl, 5 mM potassium chloride, 2.5 mM magnesium sulfate, 1 mM calcium chloride, 20 mM HEPES, pH 7.4) supplemented with dimethyl sulfoxide (DMSO) or 20 μM cytochalasin B (Sigma-Adrich), an actin polymerization inhibitor that blocks nonspecific glucose uptake, for 1 hour at 37°C. Next, the cells were incubated in 0.01 mM 2-deoxy-D-glucose [Sigma-Aldrich], 0.665 nCi/mL [1,2-^3^H]2-deoxy-D-glucose [PerkinElmer], and either DMSO or cytochalasin B (20 μM) in Krebs buffer for an additional 20 minutes at 37°C. After this time period, cells were immediately rinsed 3 times with ice-cold PBS, then lysed in 0.4 N sodium hydroxide. Base was subsequently neutralized with 0.4 N hydrochloric acid. Uptake of [^3^H]2-deoxy-D-glucose was determined by scintillation counting (Beckman Coulter). Nonspecific glucose uptake as determined by the cytochalasin B group was subtracted, and glucose uptake in pmol was normalized to protein content as determined by the BioRad DC protein assay system. Experiments were performed at least 3 times with each cell line and condition in triplicate, and data were graphed and analyzed by t-test using GraphPad Prism software.

### Viable cell proliferation assays

HTh74 and T238 cells stably expressing pQCXIP vector with or without TXNIP were plated in duplicate in 6 cm plates at 50,000 cells/plate in RPMI 1640 supplemented with 5% FBS, without antibiotics. At days 3, 5, and 7, cells were rinsed in PBS, incubated in 0.25% trypsin-EDTA, collected, and resuspended in RPMI with 5% FBS. Cells were counted via the ViCell automated cell counting system. On day 7, collected cells were subsequently lysed in EB and subjected to Western blot analysis to determine TXNIP protein expression. Experiments were performed at least 3 times, and data were combined, graphed, and analyzed by 2 way ANOVA using GraphPad Prism software.

### Invasion assays

Invasion assays with 2×10^5^ HTh74 and 1×10^5^ T238 cells stably expressing QCXIP vector with or without TXNIP were performed as previously described using BD Biocoat Matrigel invasion chambers (8 μM pore size, 24-well; BD Biosciences) [4]. Five fields per well were counted using Metamorph software (Molecular Devices), and each condition was performed in triplicate. Data from three independent experiments were combined, and data averages were normalized to the vector control mean. Statistical analysis was performed via application of the two-tailed t-test using GraphPad Prism software.

### Orthotopic tumor mouse model

The right thyroid lobes of athymic nude mice were injected with 500,000 T238 QCXIP and T238 TXNIP cells stably expressing a luciferase-IRES-GFP plasmid (pEGFP-Luc-N1, a kind gift from C. Li, University of Colorado Denver, Aurora, CO) in 5 μL PBS as previously described [4,38-41]. Weekly bioluminescence imaging using Xenogen IVIS200 (Caliper Life Sciences) in the presence of injected luciferin substrate (Caliper Life Sciences) was performed to monitor tumor establishment and growth, and bioluminescence activity was analyzed using Living Image software (Xenogen Corporation). Bioluminescence curves were analyzed by 2-way ANOVA with Bonferroni post-tests using GraphPad Prism software. There were 10–11 mice per group for each experiment, and the described experiment was performed two times. *In toto*, there were 21 mice in each experimental arm when data from the two independent studies were pooled. Animals were sacrificed at 26–28 days or sooner if ill or moribund, and final tumor dimensions were measured with calipers. Final tumor volumes were calculated using the formula (length × width × height)/0.5236 and compared with t-test using GraphPad Prism software. All procedures were conducted in accordance with a protocol approved by the Institutional Animal Care and Use Committee of the University of Colorado Denver.

### Isolation of RNA from lungs and quantitative reverse transcription polymerase chain reaction (qRT-PCR) for eGFP expression

At time of sacrifice for the second mouse orthotopic injection experiment, lungs were collected, snap-frozen in liquid nitrogen, and stored at −80°C. Lungs from uninjected mice served as negative controls. To harvest RNA, lung tissues were diced in a petri dish on ice and homogenized in TRI Reagent (Sigma Aldrich) using sterile stainless steel beads and a Qiagen TissueLyser. Homogenized tissue in TRI Reagent (1 mL) was mixed with 200 μL chloroform, centrifuged, and aqueous phase (which contains RNA) was removed. RNA purification with Qiagen RNeasy kit was then performed per the manufacturer’s instructions with the exception of an added step of column incubation with RNase-free DNase I stock solution (Qiagen) in between wash steps to remove any residual DNA. RNA was ultimately eluted with RNase free water, and RNA concentration was quantitated using a Synergy H1 microplate reader (BioTek).

GFP mRNA levels were measured by real-time qRT-PCR using an ABI Prism 7900 sequence detector (Applied Biosystems/Life Technologies). Primers and probe for GFP were designed with the assistance of the Prism 7900 sequence detection software (Primer Express, PE Applied Biosystems). The TaqMan probe was purchased from Life Technologies 5′ labeled with 6-carboxyfluorescein (FAM) and 3′-labeled with 6-caboxy-tetramethylrhodamine (TAMRA). The forward and reverse primer sequences were GFP- F 5′- CACATGGTCCTGCTGGAGTTC- 3′ and GFP- R 5′- TTGTACAGCTCGTCCATGCC- 3′ and the TaqMan fluorogenic probe sequence was 6FAM-CCGCCGCCGGGATCACTCT-TAMRA. Amplification reactions were performed in MicroAmp optical plates (Applied Biosystems/Life Technologies) in a 20 μl mix containing 1X TaqMan Buffer A (500 mM KCl, 100 mM Tris–HCl, 0.1 M EDTA, 600 nM passive reference dye ROX, pH 8.3 at room temperature), 300 μM each of dATP, dGTP, dCTP and 600 μM dUTP, 5.5 mM magnesium chloride, 900 nM forward primer, 900 nM reverse primer, 200 nM probe, 1.25 U AmpliTaq Gold DNA Polymerase and the template cDNA. Thermal cycling conditions were as follows: 2 minutes at 50°C followed by activation of TaqGold at 95°C for 10 minutes. Subsequently, 40 cycles of amplification were performed at 95°C for 15 seconds and 60°C for 1 minute. Quantities of GFP in test samples were normalized to 18 s r-RNA (PE Applied Biosystems), and the Mann Whitney test was applied to assess for statistical significance using GraphPad Prism software.

## Competing interests

The authors have no competing interests to declare.

## Authors’ contributions

JAM, WMW, and BRH conceived of experiments outlined in this report. JAM composed the manuscript, which was approved by all authors. WMW and VS generated the HTh74 cells expressing PPARγ-specific shRNA, and ACT analyzed the microarray data. JAM performed Western blot analyses, TXNIP IHC, subcloning of TXNIP into retroviral vector and transduction of ATC cell lines, glucose uptake assays, and viable cell proliferation assays. JAM and QZ performed the invasion assays. JAM, LAP, and QZ performed the orthotopic animal experiments. SBS provided pathology assessments of IHC and mouse tissues. JAM and JJS isolated RNA from lungs for metastasis determination in the orthotopic mouse experiment. All authors read and approved the final manuscript.
